# Assessment of Dietary Exposure to Ochratoxin A in Lebanese Students and Its Urinary Biomarker Analysis

**DOI:** 10.3390/toxins13110795

**Published:** 2021-11-10

**Authors:** Manar Al Ayoubi, Mohammad Salman, Lucia Gambacorta, Nada El Darra, Michele Solfrizzo

**Affiliations:** 1 Department of Nutrition and Dietetics, Faculty of Health Sciences, Beirut Arab University, Tarik El Jedidah—Beirut, P.O. Box 115020 Riad EL Solh, Beirut 1107 2809, Lebanon; mga249@student.bau.edu.lb (M.A.A.); n.aldarra@bau.edu.lb (N.E.D.); 2 Mycotoxins Department, Lebanese Agricultural Research Institute, Fanar P.O. Box 2611, Beirut 1107 2809, Lebanon; Relhage@lari.gov.lb; 3 Institute of Sciences of Food Production (ISPA), National Research Council of Italy, V. Amendola 122/o, 70126 Bari, Italy; lucia.gambacorta@ispa.cnr.it

**Keywords:** Ochratoxin A, biomarker, risk assessment, urine, duplicate diets, exposure

## Abstract

The present study investigated the dietary and urinary OTA occurrence among 44 Lebanese children. Relying on HPLC-FLD analysis, OTA was found in all the urine samples and in 46.5% and 25% of the 24 h duplicate diet and dinner samples, respectively. The means of OTA levels in positive samples were 0.32 ± 0.1 ng/g in 24 h diet, 0.32 ± 0.18 ng/g in dinner and 0.022 ± 0.012 ng/mL in urines. These values corresponded to margin of exposure (MOE) means of 7907 ± 5922 (neoplastic) and 2579 ± 1932 (non-neoplastic) calculated from positive 24 h diet, while 961 ± 599 (neoplastic) and 313 ± 195 (non-neoplastic) calculated from the urine. Since the MOE levels for the neoplastic effect were below the limit (10,000), a major health threat was detected and must be addressed as a health institutions’ priority. Besides, the wide difference between PDIs and MOEs calculated from food and urine suggests conducting further OTA’s toxicokinetics studies before using urine to measure OTA exposure.

## 1. Introduction

Ochratoxin A (OTA) has been recognized as one of the major mycotoxins contaminating a variety of food products. Cacao and its products, cereals and cereal-based products, dried fruits, nuts, pulses, spices, beans, grapefruit juice, beer, wine, herbal infusion, in addition to several products derived from animals, are the most susceptible goods to be contaminated by OTA, based on the European Food Safety Authority (EFSA) opinion [1,2].

*Penicillium verrucosum* and *P. nordicum* are two species from the Penicillium family that produce OTA, mainly known for its nephrotoxicity, while *Aspergillus ochraceus* and *A. carbonarius* are two of more than twenty OTA producing species of Aspergillus family that occur mostly in tropical, subtropical, and Mediterranean areas [3,4].

Research studies have concluded that frequent urinary tract neoplasm and the famed lethal disease that targets kidneys of south-eastern areas in Europe known as Balkan endemic nephropathy (BEN) could be consequences of the OTA presence in food [5,6]. This hypothesis was later confirmed by the Codex Alimentarius [7]. OTA was shown also to induce intestinal disorders by reducing the microbiota of the colon [8,9,10]. It was classified as Group 2B carcinogen according to IARC [11].

Due to its detrimental consequences on health, EFSA established in 2006 a tolerable weekly intake (TWI) of 120 ng/kg of body weight for OTA [12]. Meanwhile, EFSA updated OTA’s risk characterization method in 2020 by recommending the margin of exposure (MOE) approach [1].

Many studies have assessed the occurrence of OTA in food. Raad et al., (2014) assessed the dietary exposure of the adult urban Lebanese population to OTA. The findings of this study emphasized the importance of reducing the consumer’s exposure to OTA, since the analysis of 47 food items from different food groups collected in Lebanon showed that 30% was the approximate percentages of the mean dietary exposure levels to OTA compared to the respective toxicological reference values [13]. The occurrence of OTA in chocolate and its products, spices, dried fruits, meat products, cereals and their related products tea and coffee specimens, seasonings, juice drinks, and legumes commercialized in Hong Kong ranged between 12.5–100% but at levels under the EU limits [10].

OTA toxicokinetic studies showed that its elimination is very slow which enables it to accumulate in body tissues [8]. OTA can be metabolized through acid hydrolysis to OT-alpha (OTα) [14]. Moreover, it can be metabolized in rat liver to glucuronidated metabolites [15]. Despite these different forms of metabolism, the rate of OTA metabolism is low since the measured free OTA level in urine and stool was higher than its metabolites [16], noting that OTA is excreted predominantly through the urinary route [1]. Its serum half-live was about 35 days after about 20 h of rapid distribution (t1/2) [8,12,17]. Thus, its exposure is considered acute and chronic because of its long endurance in the body [5].

While the Lebanese are relying on staple food in a great percentage such as cereals which contribute 35% of the daily energy intake of adults in Beirut (the country’s capital city), the susceptibility to mycotoxin ingestion is considered high [18,19,20]. That is why it is of crucial interest to screen OTA in food consumed in Lebanon.

The assessment of human exposure to mycotoxins is conducted usually by evaluating dietary exposure. However, a second valid method is the measurement of mycotoxin biomarkers in body fluids such as urine. This alternative method was promoted by EFSA to assess mycotoxins consumption by measuring either unchanged and/or metabolic forms, looking for correlation between consumption and excretion of the same mycotoxin [2,21].

Several methods are available for the determination of OTA in food and urine samples. These methods comprise rapid immuno-based methods and methods based on HPLC with FLD or MS detection with or without immunoaffinity cleanup of crude extract [22,23,24]. For our analyses we selected method based on immunoaffinity cleanup of food extract or urine and HPLC with FLD detection for separation and measurement of OTA in purified and concentrated extract. This approach guarantee the best performances in terms of selectivity, sensitivity, accuracy and repeatability of results by using instrumentation (HPLC-FLD) available at reasonably low cost, which is important in non-rich Countries such as Lebanon.

This study aims to assess exposure to OTA in 44 Lebanese children who provided 24 h duplicate diet, separated dinner, and first-morning urine that were analyzed by HPLC-FLD after cleanup and concentration based on immunoaffinity column. The correlation between OTA exposure measured by food and urine analysis was also studied.

## 2. Results and Discussion

### 2.1. Population Characteristics

Forty-four out of fifty children carried on with their participation in this study. The remaining three pulled out of the study prior to the sampling day, while due to eating non-recommended food types or not collecting the samples, the other three participants were excluded from the study.

The 44 students were composed of 27 girls (61.4%) with a mean body weight of 53.3 kg and 17 boys (38.6%) with a mean body weight of 51.3 kg. The overall mean body weight was 52.5 kg (Table 1).

### 2.2. Performance of the Analytical Method

A good regression curve was obtained by injecting the eight calibration solutions (y = 707183x − 1153.5; R^2^ = 1). Based on recovery experiments from spiked foods and urines, mean OTA recoveries were 93% and 97%, respectively. The repeatability of results was 10 and 8%, respectively. The values of LOD and LOQ were 0.05 and 0.07 ng/g, respectively for food and 0.007 and 0.01 ng/mL, respectively for urine.

### 2.3. Occurrence of OTA in 24 h Diet

**Figure 1:**
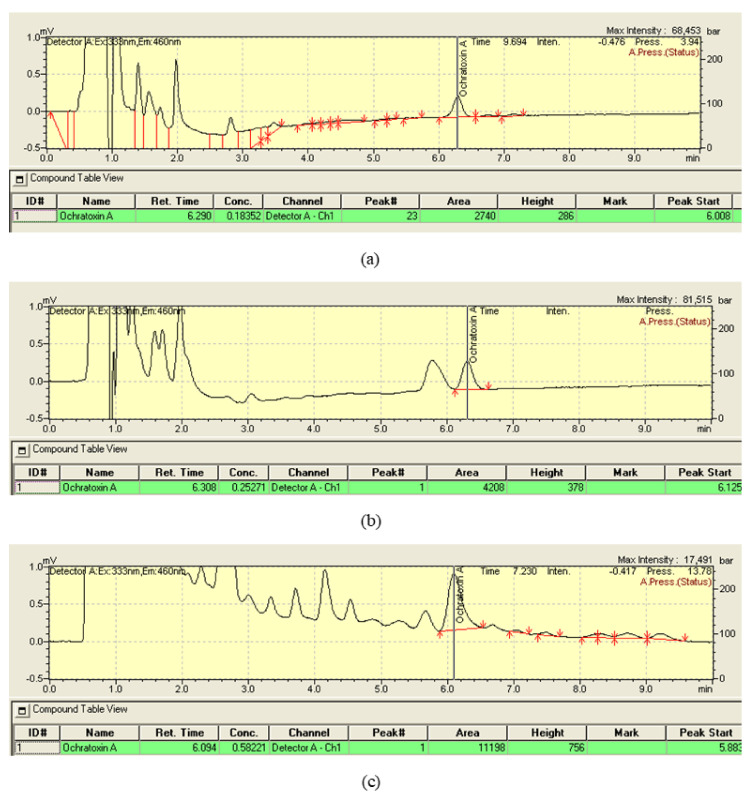
HPLC-FLD chromatograms showing OTA in (**a**) 24 h diet sample *n*. 1 (0.23 ng/g), (**b**) dinner sample *n*. 27 (0.23 ng/g), and (**c**) urine sample *n*. 41 (0.024 ng/mL).

Only the food samples of 43 out of 44 students were analyzed since one of them did not provide its 24 h diet sample. It is to be noted that the HPLC-FLD method based on immunoaffinity column cleanup is widely used for the analysis of complex food matrices [22,23]. Chromatograms showing OTA in sample extracts of a 24 h diet, dinner, and urine are reported in Figure 1.

**Figure 2:**
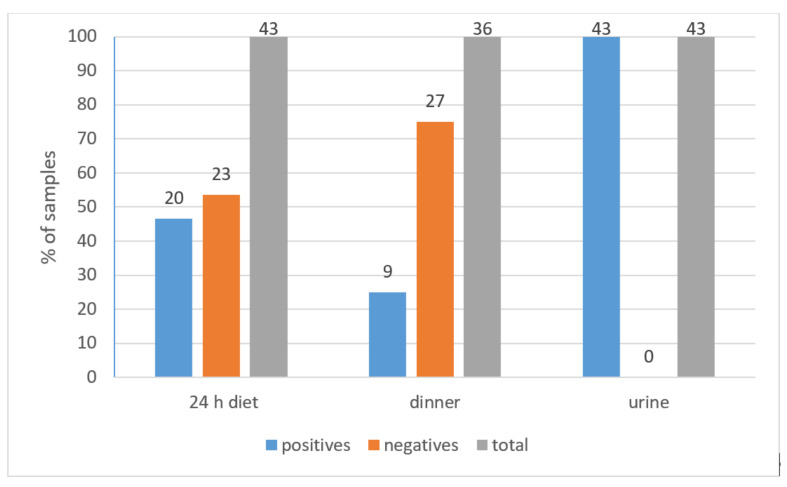
Incidence of OTA in 24 h diet, dinner, and urine samples.

While OTA was not detected in 53.5% of 24 h diet samples (23 samples) (Figure 2), a mean level of 0.32 ± 0.1 ng/g ranging between 0.18–0.55 ng/g and a median of 0.29 ng/g were found in 46.5% of the samples (20 samples) (Table 2). According to the EFSA approach, since OTA was not detected in less than 60% of samples, the samples with no detectable OTA (53.5%) were valued as half of the limit of detection of the analytical method used in this study (LOD/2 = 0.025 ng/g) [24]. In a range of 0.025–0.55 ng/g, a mean equal to 0.16 ± 0.16 ng/g and a median of 0.025 ng/g was recorded for the overall 24 h diet samples (Table 2).

To estimate dietary intake of OTA, Gilbert et al., (2001) analyzed duplicate diets over an entire month [25]. The participants in their study were adults. Even when their eaten quantity of food was higher than those of our child participants (mean 1186 g/d toward 412 g/d) (Table 1), the OTA levels of the present study are quite higher than their levels (our mean OTA 0.32 ng/g, ranging between 0.18–0.55 ng/g, is higher than their mean of 0.030 ng/g ranging between 0.010–0.115 ng/g).

### 2.4. Calculation of PDIs and MOEs from the 24 h Diet

The PDIs calculations are shown in Table 2 for the positive samples and the overall 24 h diet samples. As expected, the PDI values of positive samples (mean, median and range levels) were much higher than the values obtained for the overall samples after applying the approach of EFSA (LOD/2) on the samples negative for OTA (Table 2). Many studies have assessed OTA intake based on the 24 h diet. Among them, the mean PDI reported by Bakker et al., (2009) (4.1 ng/kg-bw) is twice as high as the 24 h diet-based PDIs of the present study [26]. Other studies have reported mean PDIs values lower than our findings i.e., 1.0 and 1.2 ng/kg-bw [27,28] (Table 3). It is important to mention that only the participants in the current study and those of Bakker et al., (2009) were children, denoting that compared to adults, due to their lower body weights, their OTA intake is higher [26].

To characterize the risk of OTA intake, the EFSA recommended, in March 2020, to apply the MOE approach after proving the invalidity of TWI [1]. The mean MOE (neo) calculated for positive 24 h diet samples was 7907 ± 5922 (79% of the limit) in a range of 2447–24344 (Table 2). The mean and range of MOE (non-neo) for these samples was 2579 ± 1932 (1290% of the limit) and 798–7941, respectively. As expected, these values increased notably for the overall 24 h diet samples after applying the EFSA approach (Nd = LOD/2), i.e., mean and range of MOE (neo) was 47342 ± 43827 (473% of the limit) and 2447-152016, respectively. For the MOE (non-neo) mean and ranges were 15443 ± 14297 (7722% of the limit) and 798–49589 (Table 2). The MOEs of the previously mentioned studies were calculated based on their reported PDIs and are shown in Table 3. Only the study of Bakker et al., (2009) had a mean MOEs (neo) below the limit (10,000) similar to the positive 24 h diet’s mean result of the present study, where their percentages of limit were 35 and 91%, respectively [26]. This indicates a health concern of neoplastic effects that OTA could cause especially for the children as shown for the child participants. While regarding the MOEs (non-neo), none of the studies had results below the limit (200).

Exposure to OTA in Lebanon is not new. In 2004, Assaf et al., stated that the Lebanese population is exposed to OTA [32]. Since the Lebanese Mediterranean diet is based on wheat-based food in addition to other cereals and pulses [19], and since it is known that these products are susceptible to OTA contamination [1,20], exposure to this mycotoxin is expected. Many Lebanese studies have assessed exposure to OTA, however, this is the first study that applied the duplicate diet approach in Lebanon. A total diet study found that the estimated mean OTA intake for adults was 4.28 ng/kg-bw [13], while a mean PDI of 5.5 ng/kg-bw for children (9–14 years old) was found by Soubra using 24 h diet recall [33]. These two studies applied the EFSA’s approach, thus comparing their PDIs with the mean PDI of the overall 24 h diet samples found in the current study (1.4 ng/kg-bw), their higher results indicate overestimations as concluded in Soubra’s studies [33,34], and prove an advantage of using duplicate diet approach in estimating OTA exposure.

Despite the mentioned advantage of the duplicate diet approach, the inability to identify the main contributor food items or meals due to their homogenization as one sample could be a limitation. Due to animal product restrictions in the present study, the studied diet can be classified as vegetarian. According to the study of Gilbert et al., (2001), a higher mean OTA intake was observed for the vegetarian participants but no differences were observed between the diets [25]. The participants in our study consumed food from all the types of studied diets, therefore, it was not possible to identify the type of food that mainly contributed to OTA intake.

Table 4 displays daily intake estimations of OTA from food items consumed in several Countries [30,35,36,37,38,39,40,41,42,43,44,45,46,47]. These food items are commonly consumed in Lebanon and by children that participated in our study. As expected, the main contributors to OTA exposure were cereals and cereal-based products. This is in accordance with EFSA who declared that cereals and cereal-based products are the main and constant contributors to OTA exposure for all age groups [1]. In addition, a Canadian study [48] and the two Lebanese studies of Soubra et al. [34] and Raad et al. [13] agreed with this statement. In particular, the Lebanese studies showed that cereal-based products contributed to up to 70% of the exposure (including pizza, bread, cakes, manakeesh, and biscuits) [34], and around 50% of the exposure (including bread and toast, biscuits and croissants, cakes and pastries, pasta and other cereal products, pizza and pies, rice) [13]. Besides the fact of OTA contamination susceptibility for these types of food, the higher daily level of consumption than the other food groups is another cause.

In comparison with the normal Lebanese diet, exposure to OTA may have been underestimated in the present study due to the restriction of animal products. This restriction, in addition to the fiber exclusion, was necessary to better link the dietary OTA consumption with its urinary excretion without dietary influence. Regarding meats and meat products, they were observed to be an important source of OTA in Egypt and the main contributor to OTA exposure in Viet Nam [49,50]. As for eggs, the incidence of OTA was found to be high in Viet Nam [50].

### 2.5. Occurrence of OTA in Dinner

Thirty-six out of the forty-four participants brought their dinner samples for analysis.

A chromatogram showing OTA in a dinner sample extract is reported in Figure 1.

While OTA was not detected in 75% of dinner samples (27 samples) (Figure 2), a mean level of 0.32 ± 0.18 ng/g, ranging from 0.18–0.76 ng/g, and a median of 0.23 ng/g were found in the remaining 25% of the samples (9 samples) (Table 2). Applying the approach of EFSA, since OTA was not detected in more than 60% but ≤80% of the dinner samples and the quantified samples were less than 25%, those having undetectable OTA were counted once as 0 and once as equal to LOD (0.05 ng/g) [24]. Means equal to 0.08 ± 0.16 ng/g and 0.12 ± 0.15 ng/g were recorded for the overall dinner samples after applying the two approaches, respectively (Table 2).

Observing the types of food eaten at dinner were kaak sprinkled with sumac (traditional Lebanese bread that can be stuffed with cheese or other food options), a sandwich of thyme mixture (dried thyme, sumac, sesame, and olive oil greased on common Lebanese white bread), or noodles—OTA was detected in some of the samples while it was absent in others. OTA was not detected at all in any of thyme manakish (Lebanese pizza topped with thyme mixture), chocolate sandwich (Lebanese white bread with spreadable chocolate), chickpeas paste sandwich, falafel with tahini sandwich (deep-fried chickpeas/fava beans balls prepared with spices and fresh herbs), halaweh sandwich (sweet tahini product), or other wheat products such as toast eaten with tea (tea herbs were also analyzed). It should be noted that in addition to the complexity of the dinner meal from the multiplicity of ingredients to the breadth of the sources, some of the students included snack food to the dinner such as popcorn, nuts, chocolate bars, and seeds such as pumpkin seeds, all of which hindered the effective comparison between dinner samples.

Bearing in mind that the dinner was analyzed twice (one was included in the 24 h diet), when comparing the results of dinner samples with those of 24 h diet samples, six dinner samples contained higher OTA levels than what was found in the corresponding 24 h diet samples. Moreover, OTA was not detected in 24 h diet samples containing four out of these six dinner samples. The range of contamination level in the six positive dinner samples was 0.18–0.76 ng/g where the highest level (0.76 ng/g) was measured in a sample of instant noodles. This packed product that includes chili spices was eaten by three participants (same brand), however, OTA was detected in two of them recording 0.41 ng/g and 0.76 ng/g. A suggestion orients the source of OTA to the spices of the packed noodles that can be supported by a recent Indonesian study, the country from where the product’s raw materials are exported from the brand’s central factory, despite its irrelevance due to the insufficient number of analyzed samples. That study showed that in a range of 23.7–84.6 ng/g, OTA contaminated 50% (3/6) of dried chili samples (whole, ground, and powder forms) [51]. In different concentrations and percentages of occurrence, OTA was found also in spices studied in Chile, Canada, Italy, Portugal, Lebanon, and Qatar, [37,48,52,53,54,55]. This could be a worrisome issue in countries such as Lebanon, where the level of spices consumption at an individual level can be as high as 5.37 g/portion, which was recorded for cinnamon in a recent study [38].

To our knowledge, this is the first study that compares OTA levels in the analyzed 24 h diet with that of dinner, thus some observations were discussed instead of comparing them with other similar studies. As mentioned above, the OTA occurrence in four dinner samples was not confirmed in the corresponding 24 h diet samples consumed by the same participants. This could be explained by the chance factor of ingredients contamination regardless of the fact of ingredients’ origin and quantity equality of the duplicate dinner. Taking an example of the dried thyme in the thyme mix of a sandwich, OTA-producing fungi can grow and produce its toxin only on the exposed part to humidity before mixing the entire quantity. OTA dilution in the 24 h diet sample after mixing dinner with other meals could be another analytical cause.

Calculating OTA intake from dinner per day, 19.59 ± 13.89 ng/d, 4.9 ± 10.87 ng/d, and 7.62 ± 9.77 ng/d were the means of intake for positive samples, overall samples with nd = 0 and overall samples with nd = LOD, respectively (Table 2).

### 2.6. Occurrence of OTA in Urine

The presence of OTA conjugates in urine has not only been confirmed by Muñoz et al., (2017) [56], but researchers are still discovering new OTA metabolites such as the newly identified ochratoxin-N-acetyl-L-cysteine (OTB-NAC) in urine [57]. Deconjugating OTA’s conjugated forms prior to the urinary-excreted OTA analysis was carried out previously by Solfrizzo et al., (2011) relying on the enzyme *β*-glucuronidase [58]. This enzymatic hydrolysis was recommended by EFSA for more effective OTA detection [1].

Forty-three out of the forty-four participants brought their first-morning urine samples for analysis. The measured urinary OTA was the sum of OTA consumed the day before and partially excreted in the following 12–24 h plus the OTA consumed in previous days and partially, but continuously, excreted in urines due to the long half-life of this mycotoxin. Figure 1 shows a chromatogram showing the peak of OTA in a urine sample.

All urine samples (43 samples) were positive for OTA with a mean concentration of 0.022 ± 0.012 ng/mL ranging between 0.007 ng/mL and 0.058 ng/mL and a median of 0.019 ng/mL (Table 2).

Similar to the present work, several studies conducted in various countries have studied OTA occurrence in human urine. Our 100% positive result was also seen in Italy [21]. The percentage of positive samples reported by other studies were 93% in Portugal [29], 87% in Bangladesh for children only [59], 100% in Korea [60], 27% in Portugal [31], and 0.1% in Sweden [61]. The exceptionally low percentage of positive samples found in the latter study was explained by the usage of an analytical method with unacceptable sensitivity.

The mean results found in the present study (0.022 ± 0.012 ng/mL) are similar to the studies of Silva et al., (2019) (0.02 ng/mL) [29] and Ahna et al., (2010) (0.031 ng/mL) [60]. However, our results were expected to be higher because we excluded from the diet probiotic and fiber-rich food which decreases the bioavailability of OTA [4,62], especially when Solfrizzo et al., (2014) in Italy and Ali and Degen (2020) in Bangladesh found mean levels much higher than ours (0.144 ng/mL for adults and 0.13 ng/mL for children, respectively) without probiotic and fiber exclusion [21,59]. The children in Ali and Degen’s study were aged 1 to 6 years, meaning that the probability of taking probiotics from milk was high. Since the Lebanese Mediterranean diet is rich in vegetables, dairy products, and fruits [63], our mean result was assumed to be lower than what was found without excluding them in the current study.

Even though the current study focused on a narrow age group (12 to 14 years old), however, it is important to mention that according to the study of Silva et al., (2019) and Akdemir et al., (2010), children’s dietary habits and the anthropometric data for both children and adults had no impact on the urinary OTA concentration [29,64].

### 2.7. Calculation of PDIs and MOEs from Urine

By applying formula 1 (mL/kg/h × 52 kg × 24 h), the daily urine output was calculated to be 1248 mL/day, where 52 kg was the mean body weight found in the present study. The urine-based PDI was then calculated with the formula reported by [21]. The 2.6% excretion rate of urinary OTA was used for PDI calculation since the inaccuracy of the 50% excretion rate reported by Heyndrickx et al., (2015) was proven in the discussion of Mitropoulou et al., (2018) [65,66]. In a range of 5.6–63.8 ng/kg bw/day, the mean PDI was 21.73 ng/kg bw (Table 2). The results of MOEs calculations showed that 961 ± 599 (10% of the limit) was the mean MOE (neo) that was in a range of 227–2585 (2–26% of the limit), while 313 ± 195 (157% of the limit) was the mean MOE (non-neo) that was in a range of 74–843 (37–422% of the limit) (Table 2). Because the application of the MOE approach for OTA has recently been recommended, we calculated the MOE for some previous studies and displayed them in Table 3 in order to compare them with those of the present study (Table 3). Almost similar results were found in Portugal by Silva et al., (2019) where their mean and maximum PDIs were 33 and 85 ng/kg-bw/d, respectively [29], and by Martins et al., (2019) where their median (5 ng/kg-bw) was within the range but lower than our (18 ng/kg-bw) [31]. However, in Southern Italy, in 2014, the mean and maximum PDIs (139 and 2047 ng/kg-bw, respectively) were much higher than ours [21]. The newly calculated means of the urinary MOEs (neo and non-neo) with the corresponding percentages of the limit for these studies ranged from 104 (1%) and 34 (17%) [21], 439 (4%) and 143 (72%) [29], 468 (5%), and 153 (76%) [30], as well as 2900 (29%) and 946 (473%) [31]. Most of the results of the studies reported in Table 3 are below the limits (10,000 and 200 for neo and non-neo, respectively) indicating a serious health concern despite that our MOEs (neo and non-neo) were slightly higher than those of the previous studies.

### 2.8. Differences between Diet-Based PDIs and Urine-Based PDI

Gilbert et al., (2001) found a wide difference between plasma clearance-based PDI and diet-based PDI and the correlation between dietary OTA intake and its concentration in urine was strong [25]. Regarding the urine-based PDI, the findings of Solfrizzo et al., (2014) showed that it was 5.8 to 127 times higher than the diet-based PDI [21]. The findings of the present study are of the same magnitude as their findings since our urine-based PDI (21.73 ng/kg-bw) is much higher than the diet-based PDI (1.4 ng/kg-bw). Non-dietary sources of OTA could be possible and were proposed by Gilbert et al., (2001) and Franco et al., (2019) [25,30] to explain the higher values of urine-based PDI. Moreover, they suggested a possible underestimation of diet-based PDI if participants did not report the consumption of some food products. This is possible when the diet-based PDI is calculated from food data obtained through interviews; however, not in our study, where we analyzed the duplicate diet. The duplicate diet approach has its advantage over other approaches in preventing such underestimations. According to Jager et al., 2014, the urine-based PDI cannot be representative when using only the first-morning urine since it does not represent the 24 h excreted volume [67]. Furthermore, because of inadequate reliable OTA human toxicokinetic information [68], the use of piglet’s excretion rate of OTA (2.6%) could overestimate urine-based PDI in humans.

### 2.9. Correlations

Possible correlations were evaluated between OTA intake (ng) obtained from the analysis of 24 h diet or dinner and OTA urinary concentration measured in the first-morning urine. LOD/2 was used for 24 h diet samples negative to OTA (53% of samples) and nd = LOD for negative samples of dinner (75% of samples).

**Figure 3:**
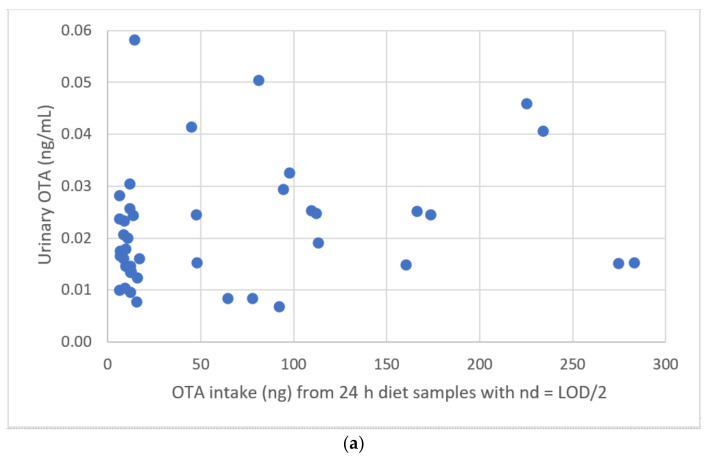

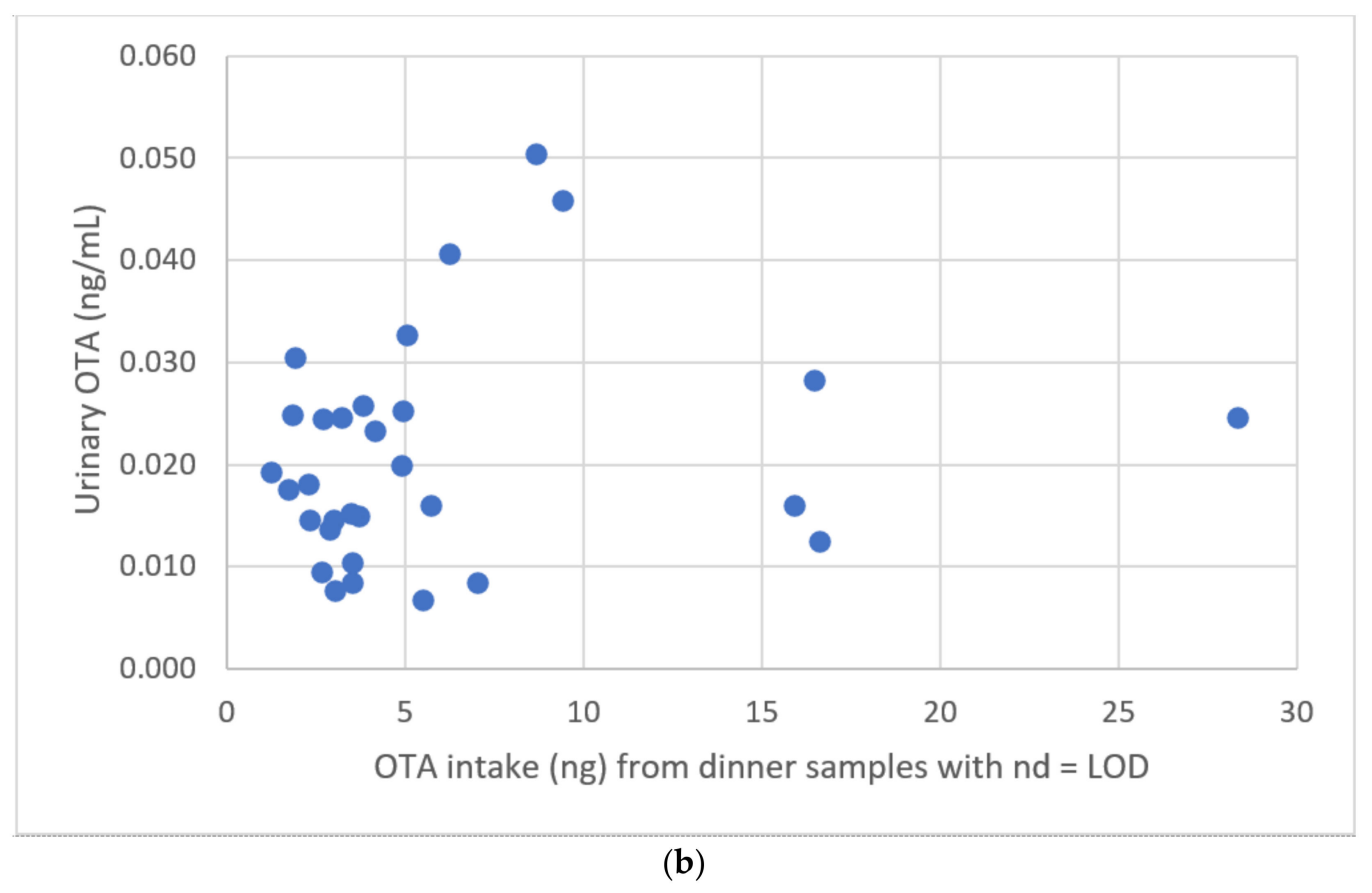
(**a**) Correlation between OTA concentrations in first morning urines and OTA intake from 24 h diet, (**b**) correlation between OTA concentration in the first morning urine and OTA intake from the dinner samples.

As shown in Figure 3, no correlation was observed between OTA intake from 24 h diets and urinary concentrations of OTA (R^2^ = 0.041). The same result was obtained by correlating OTA intake from dinners and urinary concentrations of OTA (R^2^ = 0.031). A previous study found a non-significant correlation between pork consumption and OTA occurrence in urine which was explained by its long half-life in plasma [66]. Kouadio et al., 2014 found a good correlation between cassava product consumption and occurrence (percentage of positive sample) of urinary OTA but no quantitative correlation was studied between OTA intake and urinary OTA concentrations [69]. On the other hand, a good correlation between OTA intake (ng/day) and urinary OTA concentration (ng/mL) was found by Gilbert et al., (2001) [25].

## 3. Conclusions

The activities and results reported in this paper can be summarized in the following points:

(1) 46.5% of 24 h diet samples of Lebanese children were positive for OTA with a mean of 0.32 ± 0.1 ng/g. The calculated means of PDI, MOE (neo) and MOE (non-neo) were 2.78 ± 1.65 ng/kg bw, 7907 ± 5922, and 2579 ± 1932, respectively.
(2) 25% of dinner samples were positive for OTA with a mean of 0.32 ± 0.18 ng/g and the calculated mean PDI was 4.9 ± 10.9 ng/kg bw.
(3) All urine samples were positive to OTA with a mean of 0.022 ± 0.012 ng/ml. The calculated means of PDI, MOE (neo) and MOE (non-neo) were 21.73 ± 13.8 ng/kg bw, 961 ± 599, and 313 ± 195, respectively.
(4) There was a large difference between the 24 h diet-based mean PDI (2.78 ± 1.65 ng/kg bw) and urine-based mean PDI (21.73 ± 13.8 ng/kg bw). Moreover, no correlation was found between 24 h diet-based PDIs and urinary concentrations of OTA.

The results of MOEs for the non-neoplastic effect (non-neo) was no matter of concern because all the mean values were above the limit (200) when calculated from both dietary and urinary data. Meanwhile, the calculated MOEs for the neoplastic effect (neo) of OTA from the positive 24 h diet and urine emerged as a serious health problem since the mean values were below the limit of 10,000. The wide difference between the 24 h diet-based PDI and urine-based PDI indicated the need to conduct further studies on OTA’ toxicokinetics to understand if urinary’ OTA can be or cannot be used as a valid biomarker of exposure.

## 4. Materials and Methods

### 4.1. Subject Background Information and Ethical Approval

From a private school in Tripoli city of North Lebanon, 50 healthy children, males, and females, aged 12–14 years old were asked to participate in the present study, and 44 students accepted to participate. The participation was accepted after ensuring that they did not have any metabolic disorders or liver/kidney problems. Consent forms signed by the students and their parents were collected. The protocol was ethically approved by the institutional review board (IRB) of Beirut Arab University with an approval code of 2018H-0051-HS-M-00294 (15 November 2018).

### 4.2. Sampling Design

#### 4.2.1. Education

The investigator explained and discussed the purpose of the study and the method of food selection with the students and their parents at school, through discussing on cell phones, and through distributing hard copies that contained detailed information. The target was to focus on the susceptible food groups to OTA contamination which include cereals and cereal-based products mainly, beans, cocoa, dried fruits, spices, and others [70,71,72,73,74]. It was asked that all food products that require cooking or other home processing steps be collected as raw. To ensure effective work during the extraction process at the laboratory, all meat and meat products were restricted, while milk and dairy products were banned to avoid result biases as their probiotic content act as reducing agents to the bioavailable mycotoxins. Besides the animal products, all kinds of fruits and vegetables, except for a very small quantity during meal cooking, were restricted to avoid result biases that can be caused by the effect of fiber on the bioavailability as well.

#### 4.2.2. Materials, Data Collecting Form, and Snack Distribution

For a successful sampling process, the participants received labeled materials of urine cups for urine collection and a specific plastic bag with a sealing strip for each meal. A 24 h diet recall form (to list in a table the content of each meal separately declaring the used quantity of vegetables, if any, and to specify the gender and body weight) was appended with snacks of a bar of chocolate, a tablespoon of uncooked popcorn kernels, and a piece of dried fruit as additional probable sources of OTA. The snacks were purchased from local markets in Tripoli, where the Lebanese, Turkish, and Saudi Arabian brands of chocolate (3 brands), 6 kinds of dried fruits (medium size), and the three brands of popcorn kernels were distributed randomly.

### 4.3. Samples Collection and Preparation until Extraction

Forty-four participants were selected to participate in the study and provided samples of their 24 h diet, dinner, and first-morning urine. One child did not provide urine, another did not provide their 24 h diet, and eight did not provide dinner samples. In total, we collected 43 urine, 43 24 h diet, and 36 dinner samples for a total of 122 samples. First-morning urine and food were requested to be sampled on the day following the duplicate diet. Both the food and urine samples were collected in cold containers at school on the urine sampling day and after an immediate delivery to the laboratory, they were kept at −18 °C until analysis.

Food samples were weighed, dried, and ground. The drying process was carried out in a hot air oven (105 °C). All meals for each participant were blended as one sample and then frozen at −18 °C until analysis.

### 4.4. Chemicals and Reagents

Immunoaffinity columns Ochraprep (R-BIOPHARM, Glasgow, Scotland), acetonitrile (Fluka Chemika, Buchs, Switzerland), heptane (Sigma-Aldrich, St. Louis, MO, USA), EDTA, acetic acid (BDH), phosphate-buffered saline (PBS) (Sigma-Aldrich), sodium acetate hydrate (HIMEDIA, Mumbai, India), HPLC-grade methanol (Fisher Scientific, Leicester, UK), ascorbic acid (BDH, West Yorkshire, England), β-glucuronidase enzyme (Sigma-Aldrich), and OTA standard (Trilogy^®^, Washington, MO, USA) were used in this study.

### 4.5. Preparation of Urine Samples

The first step to prepare the urine sample was thawing, followed by centrifuging at 4500 rpm for 10 min in a Falcon tube. A hydrolysis buffer was prepared to dissolve 13.6 g sodium acetate hydrate, 1.0 g ascorbic acid, and 0.1 g EDTA in 100 mL deionized water, and pH 5 was reached with acetic acid 98% and 3 mL of it was added to 5 mL of the centrifuged urine. Forty microliters of the enzyme *β*-glucuronidase >85,000 unit/mL was added to the buffered urine and the sample was kept at 37 °C overnight. A second centrifugation was carried out for the hydrolyzed sample the next day at 4500 rpm for 5 min. The hydrolyzed urine was purified using immunoaffinity columns (IAC) containing antibodies specific for OTA. The column was conditioned by passing 1 mL of distilled water that was discarded together with the buffer present in the column. The mixture of buffered hydrolyzed urine (8 mL) sample was passed through the column and discarded at a flow rate of about 1 drop/sec. The column was washed with 5 mL of distilled water at a flow rate of 1–2 drops/s and the eluate was discarded. OTA was eluted and collected in a silanized glass vial by slowly passing 3 mL of methanol HPLC grade through the column, making sure that the elution was performed until the column was dry by pressing air through it. Thereafter, at 50 °C, a gentle stream of nitrogen was used to evaporate the eluate until complete dryness. The residues were finally redissolved in 500 µL of the HPLC mobile phase (ACN: H2O: Acetic acid, 99:99:2, *v*/*v*/*v*), and stored at 4 °C until HPLC analysis. IMA cleanup and HPLC-FLD for OTA determination in urine is a widely used approach in the last 10 years in the analytical laboratories worldwide. We have introduced an enzymatic hydrolysis step in the pre-treatment of urine before IMA cleanup in order to measure the sum of free OTA and glucuronides of OTA recently confirmed in human and animal urines [56,75].

### 4.6. Preparation of Food Samples

We used the AOAC method (AOAC Official Method 2000.03) reported in the IAC’s specific manual, for the determination of OTA in food as follows: 5 g of defrosted and ground sample were vortexed with 20 mL of acetonitrile: water (60:40 *v*/*v*) extraction solvent in a Falcon tube for 5 min. The sample was then filtered using Whatman No. 2 filter paper. Five milliliters of the filtrate was vortexed for a second time for 2 min after the addition of 5 mL of heptane. A centrifugation step was subsequently performed for 10 min at 4000 rpm. To 4 mL of the bottom layer of the extract was added 44 mL of phosphate-buffered saline (PBS) solution, and 48 mL of diluted sample was loaded on a previously conditioned IAC column discarding the buffer present in the column and passing 1 mL of distilled water. After discarding the IAC eluate, 20 mL of PBS was then loaded and passed through the IAC column as a washing step. OTA was collected in a silanized glass vial by passing, at a very slow rate, 1.5 mL of acetic acid: methanol (2:98 *v*/*v*) elution solvent into the column and backflushing again, followed by loading 1.5 mL of distilled water (3 mL total volume). The purified extract was stored at 4 °C until HPLC analysis.

### 4.7. High-Performance Liquid Chromatography with Fluorescence Detection (HPLC-FLD) Analysis

HPLC analyses were performed with a Shimadzu HPLC (Shimadzu, Kyoto, Japan) consisting of a binary pump capable of producing a flow rate at 1 mL/min, a syringe loading injection valve with a 100 µL loop. Chromatographic separation was performed by a reverse-phase column (Zorbax C18, 150 mm × 4.6 mm, 5 µm particles, Phenomenex, Torrance, CA, USA) preceded by a 0.5 µm filter and thermostated at 30 °C. The mobile phase was ACN: H2O: Acetic acid (99:99:2, *v*/*v*/*v*) eluted at rate of 1 mL/min. A fluorescence detector (Shimadzu RF-20A xs) was used and set at 333 nm and 460 nm for excitation and emission wavelengths, respectively. The injection volume was 30 µL for both purified urine extracts and purified food extracts. The run time was 10 min. The analytical method used in this study is the European Standard Method EN 14132:2003 also adopted by AOAC as Official Method 2000.03. This method was successfully validated with an interlaboratory study conducted on barley but resulted equally efficient for other cereals and foods by several laboratories worldwide and recommended by companies producing immunoaffinity columns specific for OTA. Successively the European Committee for Standardization (CEN) decided to update this method by editing EN 14132:2009 who incorporated a similar method specific for roasted coffee.

### 4.8. Performance of the Analytical Methods

#### 4.8.1. Calibration Curve

An OTA standard mother solution (10 mL; 0.1 µg/mL) was prepared by diluting 0.1 mL of the OTA standard solution (10 µg/mL) with 9.9 mL of acetonitrile. Adequate volumes of this solution were used to prepare eight OTA calibration solutions. In particular, calibration solutions were prepared by diluting 0.002, 0.005, 0.01, 0.02, 0.03, 0.05, 0.1, and 0.2 mL of the OTA standard mother solution up to 1 mL with acetonitrile. The resulting eight final concentrations of OTA calibration solutions were: 0.2, 0.5, 1, 2, 3, 5, 10, and 20 ng/mL, respectively. The OTA calibration curve consisted of eight calibration points injecting 30 µL of each calibration solution.

The expression of the correspondent linear regression equation and the correlation coefficient were used to calculate OTA concentrations in the studied samples.

#### 4.8.2. Exposure Assessment (Probable Daily Intakes Estimates)

Based on the OTA concentrations found in the samples of both the 24 h duplicate diets and urine, the probable daily intake (PDI) for this mycotoxin was calculated, followed by calculating its MOE and comparing it to the correspondent limits.

#### 4.8.3. 24 h Diet Approach

Applying the previously used PDI equation by [35], OTA’s PDI based on the 24 h diet approach was calculated as follows:

*PDI* = (*C* × *K*)/*bw*. (1)

where:

- *C* is OTA level in food (ng/g)
- *K* is the weight of the consumed food (g)
- *bw* is the body weight reported by each participant (kg)

Taking into consideration that all required data for PDI calculation were individualized.

Regarding the MOE approach, the EFSA established two different BMDL10 for critical neoplastic (14.5 µg/kg-bw/day) and chronic non-neoplastic effect of OTA (4.73 µg/kg-bw/day) [1].

The equation is as follows [1]:

*MOE* = *BMDL10* (*µg*/*kg* − *bw*/*day*)/*Exposure data* (*µg*/*kg* − *bw*/*day*). (2)

In this paper, the calculated MOE based on neoplastic effect was mentioned as MOE (neo), while the calculated MOE based on non-neoplastic effect was mentioned as MOE (non-neo)

#### 4.8.4. Urinary Approach

According to the formula of [21] that was used later by [35], OTA’s PDI based on the urinary approach was calculated as follows:

*PDI* = (*C* × *V* × 100)/(*W* × *E*). (3)

where:

- *C* is the urinary OTA concentration of each participant (ng/mL)
- *V* is the normal daily urine excretion of children i.e., 1 mL/kg/hour (1250 mL/day) that is between 0.5 and 1.5 mL/kg/hour estimated by the centers of disease control and prevention (CDC) (https://www.cdc.gov/dengue/training/cme/ccm/page57297.html accessed 9 November 2021) [76]
- *W* is the body weight of each participant (kg)
- *E* is the ratio of OTA urinary excretion (2.6%) [21,77].

The normal daily urine excretion was unified for all participants by taking children’s body weight as the mean body weight found in the present study.

MOE was calculated based on OTA urinary levels as well.

#### 4.8.5. Dinner

The daily OTA exposure from dinner separately was expressed as ng/d through applying *C* × *K* where:

- *C* is OTA level in dinner (ng/g)
- *K* is the weight of dinner consumed by each participant (g)

### 4.9. Risk Characterization

The EFSA relies on two limits for MOE depending on the neoplastic and non-neoplastic effects of OTA (10,000 and 200, respectively). An MOE ≥10,000 for the chronic neoplastic effect or ≥200 for the chronic non-neoplastic effect of OTA indicates that the exposure would be of low health concern [1]. These two limits have been used in the present study to characterize the findings of food and urine samples.

### 4.10. Statistical Analysis

Minimum values, maximum values, mean ± SD values, and P95 were calculated using Microsoft Excel 2013 for each of the 24 h diet samples, dinner samples, urine samples, their PDIs, and their MOEs. To distinguish between males’ and females’ results, two-tailed *p* values were calculated. The calculation of correlation coefficients between sample types was performed. The calculations for positive samples only and for overall samples (after applying the left-censored approach) were separated.

## Figures and Tables

**Table 1:** Results of body weights, food intake, and OTA levels in positive 24 h diets, positive dinners, and urines of students participating in the study.

	**All Participants**	**Males**	**Females**	**Two-Tailed** ***p*-Value**
N. of participants	44	17 (38.6%)	27 (61.4%)	N/A
Mean body weight (kg)	52.5	51.3	53.3	0.6037
Mean of 24 h diet weight (g)	412.36	482.8	366.3	0.0041
Mean of dinner weight (g)	68.89	65.8	71.2	0.6138
		**Males**	**Females**	**Two-Tailed** ***p*-value**
Mean OTA in positive 24 h diet (ng/g)		0.33	0.31	0.7671
Mean OTA in positive dinner (ng/g)		0.3	0.33	0.8374
Mean OTA in urine (ng/mL)		0.021	0.022	0.7643

N/A: not applicable.

**Table 2:** OTA levels in the 24 h diet, dinner, and urine and calculated values of PDIs and MOEs.

	Positive 24 h Diet	All 24 h Diet ^a^	Urine	Positive Dinner	All Dinner 1 ^b^	All Dinner 2 ^c^
Mean ± SD ^e^	0.32 ± 0.1	0.16 ± 0.16 (nd = LOD/2)	0.022 ± 0.012	0.32 ± 0.18	0.08 ± 0.16 (nd = 0)	0.12 ± 0.15 (nd = LOD)
Median (range) ^e^	0.29 (0.18–0.55)	0.03 (0.03–0.55)	0.019 (0.007–0.058)	0.23 (0.18–0.76)	0 (0–0.76)	0.05 (0.05–0.76)
Mean PDI	2.78 ± 1.65	1.4 ± 1.71	21.731 ± 13.8	19.59 ± 13.89 ^d^	4.9 ± 10.87 ^d^	7.62 ± 9.77 ^d^
Median (range) PDI	2.32 (0.6–5.93)	0.32 (0.1–5.93)	17.995 (5.6–63.8)	15.89 (5.63–53.07) ^d^	0 (0–53.07) ^d^	3.88 (1.73–53.07) ^d^
Mean MOE (neo) ± SD (% of limit)	7907 ± 5922 (79)	47342 ± 43827 (473)	961 ± 599 (10)	N/A	N/A	N/A
median MOE (neo)	6254	45414	806	N/A	N/A	N/A
Range MOE (neo) (% of limit)	2447–24344 (24–243)	2447–152016 (24–1520)	227–2585 (2–26)	N/A	N/A	N/A
mean moe (non–neo) ± SD (% of limit)	2579 ± 1932 (1290)	15443 ± 14297 (7722)	313 ± 195 (157)	N/A	N/A	N/A
median MOE (non–neo)	2040	14814	263	N/A	N/A	N/A
Range MOE (non–neo) (% of limit)	798–7941 (399–3971)	798–49589 (399–24794)	74–843 (37–422)	N/A	N/A	N/A

^a^ all 24 h diet samples after applying the EFSA approach (Nd = LOD/2) on the left censored samples; ^b^ all dinner samples after applying the EFSA approach (Nd = 0) on the left censored samples; ^c^ all dinner samples after applying the EFSA approach (Nd = LOD) on the left censored samples; ^d^ values are in ng/d; ^e^ food values are expressed in ng/g and urine values are expressed in ng/mL; N/A: not applicable; PDIs are expressed in ng/kg-bw/d.

**Table 3:** Comparison of PDIs of OTA calculated from 24 h diet and urine and the calculated MOEs of this study and previous studies.

	Mean PDI (ng/kg-bw)	Mean MOE (% of Limit)	
**Diet**			
This study	1.4	Neo	47,342 (473)
		Non-neo	15,443 (7722)
Bakker et al., (2009) [26]	4.1	Neo	3537 (35)
		Non-neo	1154 (577)
Sizoo and Van Egmond (2005) [28]	1.2	Neo	12,083 (121)
		Non-neo	3942 (1971)
Bakker and Pieters (2002) [27]	1	Neo	14,500 (145)
		Non-neo	4730 (2365)
**Urine**			
This study	21.73	Neo	961 (10)
		Non-neo	313 (157)
Solfrizzo et al., (2014) [21]	139	Neo	104 (1)
		Non-neo	34 (17)
Silva et al., (2019) [29]	33	Neo	439 (4)
		Non-neo	143 (72)
Franco et al., (2019) [30]	31	Neo	468 (5)
		Non-neo	153 (76)
Martins et al., (2019) [31]	5 (median PDI)	Neo	2900 (29) ^a^
		Non-neo	946 (473) ^a^

^a^ MOE values were calculated based on the median found in that study.

**Table 4:** The contribution of certain types of food to OTA exposure (PDI) in recent studies.

Category	Food Type	PDI (EDI) ng/kg-bw/d	Population Age	Country	Reference
Grains and grain-based products (cereals)	Grains and grain-based products (cereals) ^d^	0.72 ^a^	Children (11–14)	Czech Republic	(Ostry et al., 2015) [40]
		2.215	Adults	Turkey	(Kulahi & Kabak, 2020) [41]
	Rice	0	Adults (46.6 ± 17.0)	Brazil	(Franco et al., 2019) [30]
		0.14	Adults	Turkey	(Kulahi & Kabak, 2020) [41]
		24.7 ^b^	-	Pakistan	(Iqbal et al., 2016) [42]
		0.02	Adults	Turkey	(Golge & Kabak, 2016) [43]
		0.309	10–17	Belgium	(Meerpoel et al., 2021) [39]
	Wheat	50–2170	Adults	Egypt	(Hathout et al., 2020)
	Wheat flour	0	Adults (46.6 ± 17.0)	Brazil	(Franco et al., 2019) [30]
		0.016	10–17	Belgium	(Meerpoel et al., 2021) [39]
	Wheat bread	0.21	Adults (18–75)	Portugal	(Duarte et al., 2009) [8]
		1.789	Adults	Turkey	(Kulahi & Kabak, 2020) [41]
		1.51	Adults	Morocco	(Tabarani et al., 2020) [46]
		0.85	Adults	Turkey	(Golge & Kabak, 2016) [43]
		0.136	10-17	Belgium	(Meerpoel et al., 2021) [39]
	Couscous semolina	4	Adults	Morocco	(Zinedine et al., 2017) [47]
	Semolina	0.18	Adults	Morocco	(Tabarani et al., 2020) [46]
	Pasta	0.132	Adults	Turkey	(Kulahi & Kabak, 2020) [41]
		0.25	Adults	Morocco	(Tabarani et al., 2020) [46]
	Cereal-based snacks	0.153	Adults	Turkey	(Kulahi & Kabak, 2020) [41]
	Biscuits	0.094	10–17	Belgium	(Meerpoel et al., 2021) [39]
Confectionery ^c^	Confectionery	0.1 ^a^	Children (11–14)	Czech Republic	(Ostry et al., 2015) [40]
	Chocolate	0.106	Adults	Turkey	(Kulahi & Kabak, 2020) [41]
Cocoa	Cocoa	0.015 ^a^	Children (11–14)	Czech Republic	(Ostry et al., 2015) [40]
		0.03	Adolescent (12–18)	United States	(Mitchell et al., 2017) [36]
Nuts	Nuts	0.106	Adults	Turkey	(Kulahi & Kabak, 2020) [41]
Dried fruits	Dried fruits	0.001	Children (11–14)	Czech Republic	(Ostry et al., 2015) [40]
		0.051	Adults	Turkey	(Kulahi & Kabak, 2020) [41]
	Dried dates	0.13	Adults	Tunisia	(Azaiez et al., 2015)
	Dried figs	0.01	Adolescent (12–18)	United sStates	(Mitchell et al., 2017) [36]
	Dried raisins	0.006	Adolescent (12–18)	United States	(Mitchell et al., 2017) [36]
Spices, seasoning, and legumes	Spices, seasoning and legumes	0.155 ^a^	Children (11–14)	Czech Republic	(Ostry et al., 2015) [40]
	Beans	0	Adults (46.6 ± 17.0)	Brazil	(Franco et al., 2019) [30]
	Capsicum	23.8	Adults	Chile	(Foerster et al., 2019) [30]
	Chili	0.011	Adults	Turkey	(Kulahi & Kabak, 2020) [41]
	Red chili	0.11	Adults	Lebanon	(Al Ayoubi et al., 2021) [38]
	Black pepper	0.03	Adults	Lebanon	(Al Ayoubi et al., 2021) [38]
Herbs	Tea	0.87 ^a^	Children (11–14)	Czech Republic	(Ostry et al., 2015) [40]

^a^ The mean PDI of both genders was calculated and displayed (mean in medium bound (MB), undetectable levels were replaced by LOQ/2); ^b^ Upper bound (UB) mean PDI was calculated by replacing the undetected levels by LOD and the unquantified levels by LOQ; ^c^ chocolate (bitter, milk, filled), confectionery coated with chocolate; ^d^ Grains: flour, pasta, breads, bakery products, crackers, biscuits, rice.

## Data Availability

The data presented in this study are available within the article.
